# Magneto-Thermoelastic Response in an Infinite Medium with a Spherical Hole in the Context of High Order Time-Derivatives and Triple-Phase-Lag Model

**DOI:** 10.3390/ma15186256

**Published:** 2022-09-08

**Authors:** Ashraf M. Allehaibi, Ashraf M. Zenkour

**Affiliations:** 1 Department of Mathematics, Jamoum University College, Umm Al-Qura University, Jamoum, Makkah 21955, Saudi Arabia; 2 Department of Mathematics, Faculty of Science, King Abdulaziz University, P.O. Box 80203, Jeddah 21589, Saudi Arabia; 3 Department of Mathematics, Faculty of Science, Kafrelsheikh University, Kafrelsheikh 33516, Egypt

**Keywords:** CTE, L–S and G–N models, spherical hole, multi-phase-lag

## Abstract

The article presents the interactions of magneto-thermoelastic effects in an isotropic material with a spherical cavity. The spherical cavity is expected to be tractionless and subjected to both heat and magnetic fields. The motion equation contains the Lorentz force. Laplace’s transformation methodology is used with a refined multi-time-derivative triple-phase-lag thermoelasticity theory to develop the generalized magneto-thermoelastic coupled solution. Many results were obtained to serve as benchmarks for future comparisons. The effects of time, magnetic field, and electric permittivity under the thermal environment were investigated.

## 1. Introduction

It is largely recognized that the theory of coupled thermoelasticity deals with the physical restriction that the thermal signal propagates at infinity. Biot [1] cultivated the theory of classical thermoelasticity based upon irreversible thermodynamic guidelines. The formula of motion within this idea is hyperbolic, while the formula of heat transmission is parabolic. As a result of the inclusion of a parabolic-type heat transmission formula, the theory still experiences the uncoupled theory flaw. Lord and Shulman [2] introduced the initial generalized thermoelasticity concept, through which the Fourier regulation of heat convection is substituted by the Maxwell–Cattaneo equation, which declares a single relaxation time in Fourier’s regulation. Similarly, Green and Lindsay [3] (G–L) provided a temperature-dependent concept with dual relaxation times stated in the constitutive equations for stresses and entropy. Green and Naghdi [4,5,6] introduced three further models dubbed G–N types I, II, and III. The linearized form of the G–N type I model corresponds to the conventional thermoelasticity theory. The internal production rate of entropy is assumed to be zero in the G–N type II model, which does not include heat dissipation. The G–N type III model incorporates both prior models, as well as energy dissipation and the admission of damped thermoelastic waves. Hetnarski and Ignaczak [7] provided yet another generalized theory. This model is defined by a nonlinear field equation system based on the low-temperature thermoelasticity hypothesis. Tzou [8] and Chandrasekhariah [9,10] enhanced the dual-phase-lag (DPL) hypothesis. Tzou [8] stated that Fourier’s regulation is substituted through an approximation to modify Fourier’s law with a pair of distinct delay times for heat motion and temperature gradient. Thermoelasticity with three-phase-lag is one of the most recent advancements in thermoelasticity theory (Roy Choudhari [11]). Here, a phase lag for the thermic displacement gradient is added to the phase lags for the heat motion vector and the temperature gradient. Quintanilla and Racke [12] addressed some remarks on the theory of heat transmission models using TPL. Quintanilla [13] studied Roy Choudhuri’s [11] previous work and discovered an ill-posed issue. To solve the issue, he integrated the constitutive association along with the two-temperature heat conduction concept. El-Karamany and Ezzat [14] studied a specific heat transmission law as well as a heat transportation formula to interact with a single-phase-lag (SPL) G–N type III, a DPL G–N type II, and a DPL G–N type III. Zenkour [15,16,17,18,19,20] recently published a refined multi-phase-lag model (RPL) that has various applications. He studied many variations of the refined model to handle thermoelastic reactions of various constructions. Mukhopadhyay et al. [21] presented a three-phase-lag model of solutions for the theory of generalized thermoelasticity. Mukhopadhyay and Kumar [22] examined the impact of three-phase-lag on thermoelastic interactions in a limitless medium with a cylindrical hole caused by a thermic shock at the boundary. Mukhopadhyay and Kumar [23] investigated the results of three-phase-lag on wave propagation in a thick plate with an axisymmetric temperature distribution. Knopoff [24] and Chadwick [25] laid the groundwork for magnetoelasticity, which was further refined by Kaliski and Petykiewicz [26] and Chiriţ [27] that offered several higher-order TPL heat transfer model approximations. In [28,29,30,31], the authors show a variety of applications involving heat differential equations. Prasad [32] discussed wave propagation in a homogeneous, isotropic, and boundless material produced by a continuous line heat source using the thermoelasticity theory and three-phase-lag. Zenkour and El-Mekawy [33] examined the key features of the dual- and three-phase-lag heat-conducting equations and their implications for the understanding of coupled thermoelasticity models. Biswas et al. [34] used three-phase-lag and generalized thermoelasticity theory to handle different heat source reactions in a transversely isotropic hollow cylindrical tube in a magnetic field with rotating behavior.

In the current paper, the magneto-thermoelastic exchanges in an unbounded body with a spherical hole are analyzed using multi-time-derivative thermoelasticity theories. A refined TPL model is utilized for this objective. The Laplace transforms method [35] over time-space is used to derive the governing equation in the theoretical form. The given equations are solved and carried by the inverse of the Laplace transforms to determine the mathematical results. The results are compared numerically as well as graphically with other models and without magnetic field effects.

## 2. Formulation of the Problem

**Figure 1:**
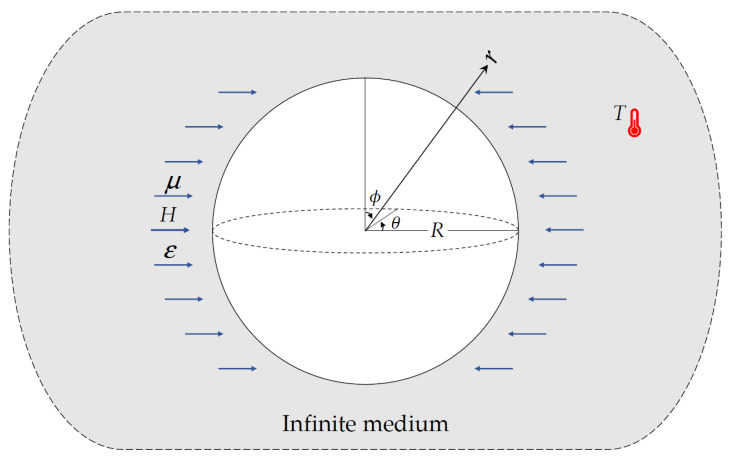
An infinite environment with constant heat and magnetic field that affect externally on the spherical hole.

An isotropic body with a spherical cavity of radius R can be analyzed using the unified multi-phase-lag theory for thermoelastic analysis. The edge of the spherical hole is considered to be traction-free and subject to constant heat (see Figure 1). The problem at hand can be solved using the spherical coordinate system (r,θ,ϕ).

The isotropic medium is considered to have a spherical hole with a radius R and an introductory uniform temperature $T_{0}$. We assumed this environment to be a symmetric thermal space; therefore, the displacement components and the temperature are defined as

(1) $$\Theta =\Theta \left(r,t\right)\begin{array}{cc} , & u_{r}=u\left(r,t\right) \end{array},\;u_{\theta }=u_{\phi }=0,$$

where Θ represents the temperature and $u_{r}$, $u_{\theta }$, and $u_{\phi }$ are the displacement components.

The non-vanishing strains can be stated as

(2) $$e_{rr}=\frac{\partial u}{\partial r},\;e_{\theta \theta }=e_{\phi \phi }=\frac{u}{r},$$

where $e_{rr}$, $e_{\theta \theta }$, and $e_{\phi \phi }$ are the strain components and the dilatation e can be expressed as

(3) $$e=e_{rr}+e_{\theta \theta }+e_{\phi \phi }=\frac{\partial u}{\partial r}+\frac{2u}{r}=\frac{1}{r^{2}}\frac{\partial \left(r^{2}u\right)}{\partial r}.$$

The constitutive equations are given by the formula

(4) $$\sigma_{ij}=2\mu e_{ij}+\left(\lambda e_{kk}-\gamma \Theta \right)\delta_{ij},$$

where $\sigma_{ij}$ represents stress tensor components, $\delta_{ij}$ represents Kronecker’s delta, $\gamma =\left(3\lambda +2\mu \right)\alpha_{t}$ represents the thermal modulus, $\alpha_{t}$ is the thermal expansion coefficient, and λ and μ are Lame’s constants.

Thus, by substituting Equation (2) into Equation (4), it gives

(5) $$\sigma_{rr}=\left(2\mu +\lambda \right)\frac{\partial u}{\partial r}+2\lambda \frac{u}{r}-\gamma \Theta ,$$

(6) $$\sigma_{\theta \theta }=\sigma_{\phi \phi }=\lambda \;\frac{\partial u}{\partial r}+\left(2\;\mu +2\;\lambda \right)\frac{u}{r}-\gamma \Theta .$$

The linear isotropic homogeneous thermoelastic body is governed by the following equation where volume forces are absent in this situation:

- The equation of motion

The equation of motion in spherical polar coordinates is introduced as a stress equation with the following form

(7) $$\sigma_{rr,r}+2\frac{\sigma_{rr}-\sigma_{\theta \theta }}{r}+\mu_{0}H_{0}^{2}\left(\frac{\partial e}{\partial r}-\varepsilon_{0}\mu_{0}\frac{\partial^{2}u}{\partial t^{2}}\right)=\rho \frac{\partial^{2}u}{\partial t^{2}},$$

where $\mu_{0}$ is electric permeability, $H_{0}$ is an initial magnetic field, $\varepsilon_{0}$ is electric permittivity, and ρ is density.

Introducing the Laplacian operator in spherical coordinates by the form

(8) $$\nabla^{2}\left(*\right)=\frac{\partial^{2}\left(*\right)}{\partial r^{2}}+\frac{2}{r}\frac{\partial \left(*\right)}{\partial r}=\frac{1}{r^{2}}\frac{\partial }{\partial r}\left(r^{2}\frac{\partial \left(*\right)}{\partial r}\right).$$

Hence, by applying Equations (3), (5), and (6) to Equation (7), one obtains

(9) $$\left(\lambda +2\mu +\mu_{0}H_{0}^{2}\right)\left(\nabla^{2}u-\frac{2\;u}{r^{2}}\right)-\gamma \frac{\partial \Theta }{\partial r}=\left(\rho +\varepsilon_{0}\mu_{0}^{2}H_{0}^{2}\right)\frac{\partial^{2}u}{\partial t^{2}}.$$

- The heat conduction equation

The heat conduction equation, in the context of the refined thermoelasticity form, is represented by a hyperbolic form as [36]

(10) $$\left(L_{T}^{*}K^{*}+L_{T}K\right)\nabla^{2}\Theta =L_{q}\left[\rho C_{\vartheta }\Theta +\gamma T_{0}\left(\frac{\partial u}{\partial r}+2\frac{u}{r}\right)\right],$$

where $L_{T}^{*}$, $L_{T}$, and $L_{q}$ are higher-order time-derivative operators given by

(11) $$L_{T}^{*}=1+\sum_{n=1}^{N}\frac{\tau_{T}^{n}}{n!}\frac{\partial^{n}}{\partial t^{n}},\;L_{T}=\left(1+\sum_{n=1}^{N}\frac{\tau_{\vartheta }^{n}}{n!}\frac{\partial^{n}}{\partial t^{n}}\right)\frac{\partial }{\partial t},\;L_{q}=\left(\varrho +\sum_{n=1}^{N}\frac{\tau_{q}^{n}}{n!}\frac{\partial^{n}}{\partial t^{n}}\right)\frac{\partial^{2}}{\partial t^{2}}.$$

and $C_{\vartheta }$ is specific heat.

Since N is an integer greater than zero; thus, Equation (10) is more generic. As a result, it gives some unique circumstances, such that

(i) Coupled thermoelasticity (CTE) model [1]: $\tau_{\vartheta }=\tau_{\theta }=\tau_{q}=0$, $K^{*}=0$, and ϱ=1,

(12) $$K\nabla^{2}\Theta =\frac{\partial }{\partial t}\left[\rho C_{\vartheta }\Theta +\gamma T_{0}\left(\frac{\partial u}{\partial r}+2\frac{u}{r}\right)\right].$$

(ii) Lord and Shulman (L–S) model [2]: $\tau_{T}=\tau_{\vartheta }=0$, $\tau_{q}=\tau_{0}$, $K^{*}=0$, N=1, and ϱ=1,

(13) $$K\nabla^{2}\Theta =\left(1+\tau_{q}\frac{\partial }{\partial t}\right)\frac{\partial }{\partial t}\left[\rho C_{\vartheta }\Theta +\gamma T_{0}\left(\frac{\partial u}{\partial r}+2\frac{u}{r}\right)\right].$$

(iii) Green and Naghdi (G–N) model without energy dissipation [4,5,6]: $\tau_{T}=\tau_{\vartheta }=0$, $\tau_{q}=0$, N=1, and ϱ=1,

(14) $$\left(K^{*}+K\frac{\partial }{\partial t}\;\right)\nabla^{2}\Theta =\left(\frac{\partial^{2}}{\partial t^{2}}\right)\left[\rho C_{\vartheta }\Theta +\gamma T_{0}\left(\frac{\partial u}{\partial r}+2\frac{u}{r}\right)\right].$$

(iv) Simple generalized thermoelasticity theory with triple-phase-lag (Simple TPL-GN theory): $\tau_{q}\geq \tau_{\theta }>\tau_{\vartheta }>0$, N=1, and ϱ=1,

(15) $$\left[K^{*}\left(1+\tau_{T}\frac{\partial }{\partial t}\right)+K\left(1+\tau_{\vartheta }\frac{\partial }{\partial t}\right)\frac{\partial }{\partial t}\right]\nabla^{2}\Theta =\left(1+\tau_{q}\frac{\partial }{\partial t}\right)\frac{\partial^{2}}{\partial t^{2}}\left[\rho C_{\vartheta }\Theta +\gamma T_{0}\left(\frac{\partial u}{\partial r}+2\frac{u}{r}\right)\right].$$

(v) Refined generalized thermoelasticity theory with triple-phase-lag (Refined TPL-GN theory): $\tau_{q}\geq \tau_{T}\geq \tau_{\vartheta }>0$, N>1, and ϱ=1

(16) $$\left[K^{*}\left(1+\sum_{n=1}^{N}\frac{\tau_{T}^{n}}{n!}\frac{\partial^{n}}{\partial t^{n}}\right)+K\left(1+\sum_{n=1}^{N}\frac{\tau_{\vartheta }^{n}}{n!}\frac{\partial^{n}}{\partial t^{n}}\right)\frac{\partial }{\partial t}\right]\nabla^{2}\Theta =\left(1+\sum_{n=1}^{N}\frac{\tau_{q}^{n}}{n!}\frac{\partial^{n}}{\partial t^{n}}\right)\frac{\partial^{2}}{\partial t^{2}}\left[\rho C_{\vartheta }\Theta +\gamma T_{0}\left(\frac{\partial u}{\partial r}+2\frac{u}{r}\right)\right],$$

where K represents heat conductivity, $K^{*}$ represents the rate of thermal conductivity, $\tau_{q}$ represents the phase lag of the heat flux, $\tau_{T}$ represents the phase lag of the temperature gradient, and $\tau_{\vartheta }$ represents the phase lag of the thermal displacement.

## 3. Closed-Form Solution

It is suitable to present the following dimensionless quantities:

(17) $$\begin{array}{c} \left\{r^{\prime },u^{\prime }\right\}=c_{0}\eta \left\{r,u\right\},\;\left\{t^{\prime },\tau_{T}^{\prime },\tau_{q}^{\prime },\tau_{\vartheta }^{\prime }\right\}=\eta c_{0}^{2}\left\{t,\tau_{T},\tau_{q},\tau_{\vartheta }\right\},\\ \sigma_{ii}^{\prime }=\frac{\sigma_{ii}}{\lambda +2\mu },\;\Theta^{\prime }=\frac{\gamma \Theta }{\lambda +2\mu }, \end{array}$$

where

(18) $$c_{0}^{2}=\frac{\lambda +2\mu }{\rho },\;\eta =\frac{\rho C_{\vartheta }}{K}.$$

All of the governing equations are reformulated using the aforesaid dimensionless variables (removing the dashes for the sake of simplicity)

(19) $$\;e=\frac{\partial u}{\partial r}+\frac{2u}{r},$$

(20) $$\sigma_{rr}=\frac{\partial u}{\partial r}+2\;\frac{\lambda }{\left(\lambda +2\mu \right)}\frac{u}{r}-\Theta ,$$

(21) $$\sigma_{\theta \theta }=\sigma_{\phi \phi }=\frac{\lambda }{\lambda +2\mu }\frac{\partial u}{\partial r}+\frac{2\left(\mu +\lambda \right)}{\left(\lambda +2\mu \right)}\frac{u}{r}-\Theta ,$$

(22) $$\frac{\mu_{0}H_{0}^{2}+\rho c_{0}^{2}}{\left(\rho +\varepsilon_{0}\mu_{0}^{2}H_{0}^{2}\right)c_{0}^{2}}\left(\frac{\partial^{2}u}{\partial r^{2}}+\frac{2}{r}\frac{\partial u}{\partial r}-\frac{2}{r^{2}}u\right)-\frac{\rho }{\rho +\varepsilon_{0}\mu_{0}^{2}H_{0}^{2}}\frac{\partial \Theta }{\partial r}=\frac{\partial^{2}u}{\partial t^{2}},$$

and

(23) $$\left(c_{T}^{2}\;L_{T}^{*}+c_{K}^{2}L_{T}\right)\nabla^{2}\Theta =L_{q}\left(\Theta +\varepsilon \nabla u\right),\;$$

where

(24) $$c_{T}^{2}=\frac{K^{*}}{\rho C_{\vartheta }c_{0}^{2}},\;c_{K}^{2}=\frac{\eta \;K}{\rho C_{\vartheta }},\;\varepsilon =\frac{\gamma^{2}T_{0}}{\rho C_{\vartheta }\left(\lambda +2\mu \right)}.$$

Equations (22) and (23) are resolved to obtain temperature Θ and radial displacement u, which are the first two variables needed to complete the set of complete solutions. Then, the subsequent volumetric strain (dilatation) e and thermal stresses are possible to display as expressions in Θ and u. To achieve this objective, the following initial conditions are applied

(25) $$u=\frac{\partial^{n}u}{\partial t^{n}}=0,\Theta =\frac{\partial^{n}\Theta }{\partial t^{n}}=0,\;\mathrm{for}\;R\leq r<\infty ,\;t=0,\;\mathrm{and}\;n=1,\ldots ,N.$$

The thermomechanical boundary conditions were also employed in conjunction with the homogeneous initial conditions. The surface of the spherical cavity is considered to be constantly heated and traction-free and the current unbounded body will be investigated as such. It is possible to describe these conditions as:

- Continuous heat is applied to the spherical hole’s outer surface

(26) $$\Theta \left(R,t\right)=\Theta_{0}\;H\left(t\right),\;t>0.$$

where $\Theta_{0}$ is thermal constant, and H(t) represents the Heaviside unit step function.

- Due to the lack of traction on the hole’s surface, the mechanical boundary condition is met

(27) $$\sigma_{rr}\left(R,t\right)=\sigma_{\theta \theta }\left(R,t\right)=0,\;t>0.$$

In addition, the following regularity requirements are taken into account:

(28) u(r,t)=0, Θ(r,t)=0, r→∞.

With the homogeneous initial conditions mentioned in Equation (24), the Laplace transform

(29) $$\overset{¯}{\psi }\left(s\right)=ℒ\left\{\psi \left(t\right)\right\}=\int_{0\;}^{\infty }e^{-st}\psi \left(t\right)dt,$$

is used on Equations (19)–(23), and the results are as follows:

(30) $$\bar{e}=\frac{d\bar{u}}{dr}+\frac{2\bar{u}}{r},$$

(31) $${\bar{\sigma }}_{rr}=\frac{d\bar{u}}{dr}+2\;\frac{\lambda }{\left(\lambda +2\mu \right)}\frac{\bar{u}}{r}-\overset{¯}{\Theta },$$

(32) $${\bar{\sigma }}_{\theta \theta }={\bar{\sigma }}_{\phi \phi }=\frac{\lambda }{\lambda +2\mu }\frac{d\bar{u}}{dr}+\frac{2\left(\mu +\lambda \right)}{\left(\lambda +2\mu \right)}\frac{\bar{u}}{r}-\overset{¯}{\Theta },$$

(33) $$\frac{\mu_{0}H_{0}^{2}+\rho \;c_{0}^{2}}{\left(\rho +\varepsilon_{0}\mu_{0}^{2}H_{0}^{2}\right)c_{0}^{2}}\left(\frac{d^{2}\bar{u}}{dr^{2}}+\frac{2}{r}\frac{d\bar{u}}{dr}-\frac{2}{r^{2}}\bar{u}\;\right)-\frac{\rho }{\rho +\varepsilon_{0}\mu_{0}^{2}H_{0}^{2}}\frac{d\overset{¯}{\Theta }}{dr}=s^{2}\bar{u},$$

(34) $$\left(\nabla^{2}-\varpi \right)\overset{¯}{\Theta }-\varpi \varepsilon \left(\frac{d\bar{u}}{dr}+\frac{2\bar{u}}{r}\right)=0,$$

where

(35) $$\varpi =\frac{{\overset{¯}{L}}_{q}}{c_{T}^{2}\;{\overset{¯}{L}}_{T}^{*}+c_{k}^{2}{\overset{¯}{L}}_{T}},$$

and

(36) $$\begin{array}{ccc} {\overset{¯}{L}}_{T}^{*}=1+\sum_{n=1}^{N}\frac{\tau_{T}^{n}}{n!}s^{n}, & {\overset{¯}{L}}_{T}=\left(1+\sum_{n=1}^{N}\frac{\tau_{\vartheta }^{n}}{n!}s^{n}\right)s, & {\overset{¯}{L}}_{q}=\left(\varrho +\sum_{n=1}^{N}\frac{\tau_{q}^{n}}{n!}s^{n}\right)s^{2} \end{array},$$

in which s represents the Laplace parameter.

The coupled system of Equations (33) and (34) can easily give the displacement $\bar{u}$ in terms of the higher derivatives of $\overset{¯}{\Theta }$ as

(37) $$\bar{u}\left(r\right)=b_{2}\;\frac{d^{3}\overset{¯}{\Theta }}{dr^{3}}-b_{2}\left(\frac{2}{r^{2}}+\varpi \right)\frac{d^{2}\overset{¯}{\Theta }}{dr^{2}}+\left(b_{1}+\frac{2b_{2}}{r}\right)\frac{d\overset{¯}{\Theta }}{dr},$$

where

(38) $$b_{1}=\frac{-\varpi \;\varepsilon \;\rho \;c0^{2}}{\Delta },\;b_{2}=\frac{H_{0}^{2}\mu_{0}+\lambda +2\;\mu }{\Delta },\;\Delta =c_{0}^{2}\left(H_{0}^{2}\mu_{0}^{2}\varepsilon_{0}+\rho \right)s^{2}\varpi \varepsilon .$$

In addition, the differential equation of temperature is expressed as

(39) $$\left(\nabla^{4}-\beta_{1}\nabla^{2}+\beta_{0}\right)\overset{¯}{\Theta }\left(r\right)=0,$$

where the coefficients $\beta_{i}$ are given by

(40) $$\beta_{0}=\frac{1}{\varepsilon \;b_{2}},\;\beta_{1}=\frac{-b_{1}\varpi +b_{2}\;\left(\varpi^{2}+\beta_{0}\right)}{b_{2}\varpi }.$$

Equation (39), which is presented in a polar coordinate system, is difficult to solve because of this. It can be stated as follows:

(41) $$\left(\nabla^{2}-\zeta_{1}^{2}\right)\left(\nabla^{2}-\zeta_{2}^{2}\right)\overset{¯}{\Theta }\left(r\right)=0,$$

where $\zeta_{j}^{2}$ are the roots of

(42) $$\zeta^{4}-\beta_{1}\zeta^{2}+\beta_{0}=0.$$

These roots are given, respectively, by

(43) $$\zeta_{1,2}^{2}=\frac{1}{2}\left(\beta_{1}\pm \sqrt{\beta_{1}^{2}-4\beta_{0}}\right).$$

Equation (41) tends to the next modified Bessel equation of zero-order

(44) $$\left(\frac{1}{r}\frac{\partial }{\partial r}\left(r\frac{\partial }{\partial r}\right)-\zeta_{1}^{2}\right)\left(\frac{1}{r}\frac{\partial }{\partial r}\left(r\frac{\partial }{\partial r}\right)-\zeta_{2}^{2}\right)\overset{¯}{\Theta }\left(r\right)=0,$$

which can be solved with the regularity condition: $\bar{u},\;\overset{¯}{\Theta }\to 0$ as r→∞. Consequently, the general solution of Equation (41), which is bounded at infinity, is provided by

(45) $$\overset{¯}{\Theta }\left(r\right)=\frac{1}{r}\sum_{j=1}^{2}A_{j}e^{-\zeta_{j}r},$$

By substituting it into Equation (37), hence $\bar{u}$, taking into account the regularity condition: $\bar{u}\to 0$ as r→∞, is given by

(46) $$\bar{u}\left(r\right)=\frac{1}{r}\sum_{j=1}^{2}{\hat{\zeta }}_{j}\left(\zeta_{j}+\frac{1}{r}\right)A_{j}{\;e}^{-\zeta_{j}r},$$

where $A_{j}$ are integration parameters and

(47) $${\hat{\zeta }}_{j}=\left(-\zeta_{j}^{2}+\varpi \right)b_{2}-2b_{1}.$$

Using the relation between $\bar{u}$ and $\bar{e}$ to obtain

(48) $$\bar{e}\left(r\right)=-\frac{1}{r\;}\overset{2}{\underset{j=1}{\sum }}{\hat{\zeta }}_{j}\zeta_{j}^{2}\;A_{j}e^{-\zeta_{j}r}.$$

The problem has been solved up to this point. The boundary conditions from Equations (25) and (26) are sufficient to determine the two parameters $A_{j}$. If the stresses are expressed in terms of radial displacement and temperature, then it is simple to give these values as

(49) $${\bar{\sigma }}_{rr}=\frac{-1}{r^{2}\left(\lambda +2\mu \right)\;}\sum_{j=1}^{2}\left[\left(\lambda +2\mu \right)\;\left({\hat{\zeta }}_{j}\;\zeta_{j}^{2}+1\right)r+4\;\mu \;{\hat{\zeta }}_{j}\left(\zeta_{j}+\frac{1}{r}\right)\right]A_{j}e^{-\zeta_{j}r},$$

(50) $${\bar{\sigma }}_{\theta \theta }=\frac{-1}{r^{2}\left(\lambda +2\mu \right)}\sum_{j=1}^{2}\left[\left(\lambda \left({\hat{\zeta }}_{j}\;\zeta_{j}^{2}+1\right)+2\mu \right)\;r-2\;\mu \;{\hat{\zeta }}_{j}\left(\zeta_{j}+\frac{1}{r}\right)\right]A_{j}e^{-\zeta_{j}r}.$$

Laplace-domain analytical solutions for the modified generalized G–N theory already exist. To obtain the solutions in the physical realm, the function ψ(t) is viewed as an inversion of the Laplace transform $\overset{¯}{\psi }\left(s\right)$ using the formula

(51) ψ(t)=ℒ−1{ψ(s)}=eqtt[12ψ¯(q)+Re(∑l=1L(−1)iψ¯(q+ilπt)) ],

where L is a sufficiently large integer, i denotes the imaginary number unit, Re represents the real part, and q is an arbitrary constant. To speed up computations, there have been numerous numerical studies demonstrating that the estimate of q that satisfies the relation is qt≈4.7 [37]. An inversion of the expressions of temperature Θ, radial displacement u, dilatation e, radial stress $\sigma_{rr}$, and hoop stress $\sigma_{\theta \theta }$ can be achieved using the numerical approach mentioned.

## 4. Validation

Numerous examples are presented to illustrate the effect of several models on the field variables. The material properties of the infinite medium with a spherical cavity are

$$\lambda =7.76\times 10^{10}{\;N\;m}^{-2},\;\mu =3.86\times 10^{10}{\;N\;m}^{-2},\;C_{\vartheta }=383.1{\;J\;\mathrm{kg}}^{-1}{\;K}^{-1},\;\alpha_{t}=1.78\times 10^{-5}{\;K}^{-1},\;\rho =8954{\;\mathrm{kg}\;m}^{-3},\;T_{0}=293\;K,\;c_{T}=1,\;c_{K}=0.697362.$$

Numerical results were obtained (except where otherwise indicated) for $\Theta_{0}=10$, $\tau_{q}=0.03$, $\tau_{T}=0.021$, $\tau_{\vartheta }=0.015$, t=0.3, and R=1.

### 4.1. First Justification

**Figure 2:**
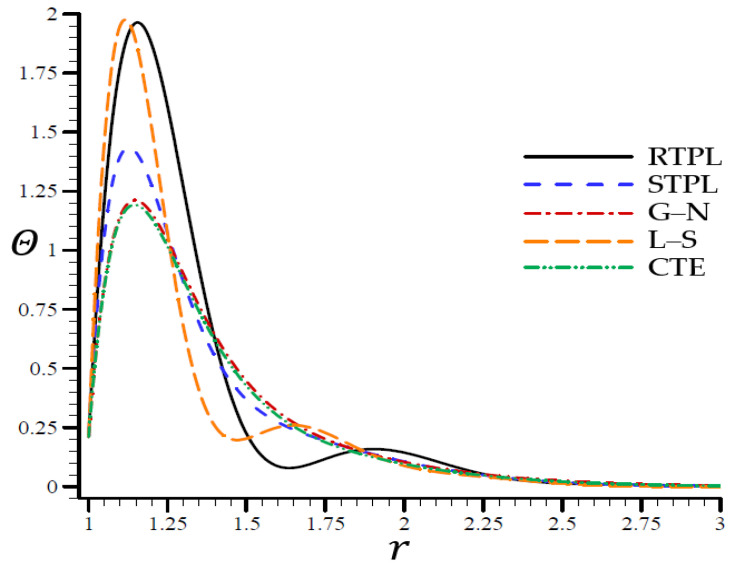
The temperature Θ through the radial direction of the spherical hole for all models.

**Figure 3:**
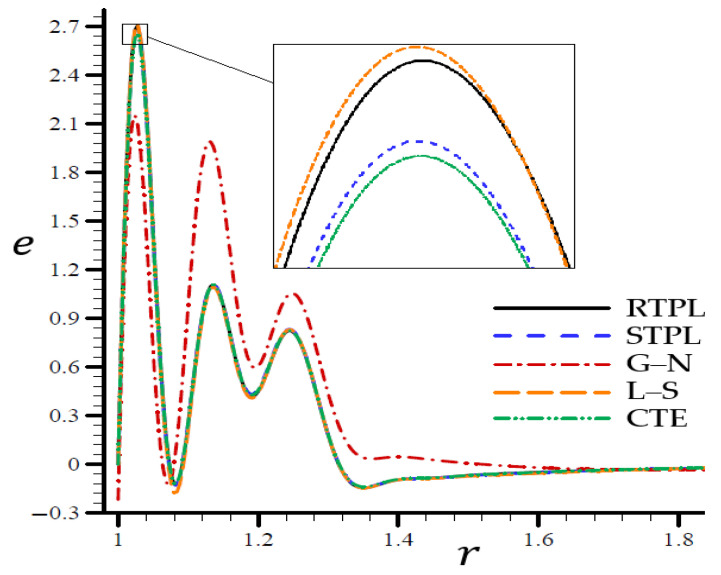
The volumetric strain e through the radial direction of the spherical hole for all models.

**Figure 4:**
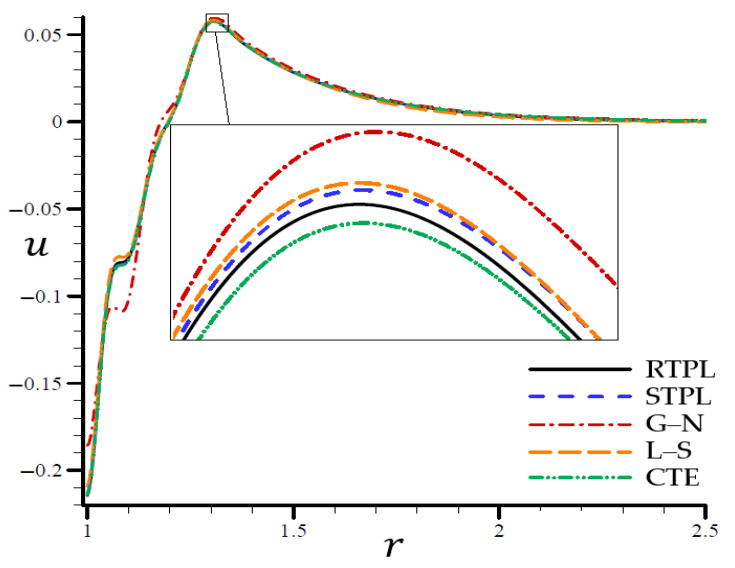
The radial displacement u through the radial direction of the spherical hole for all models.

**Figure 5:**
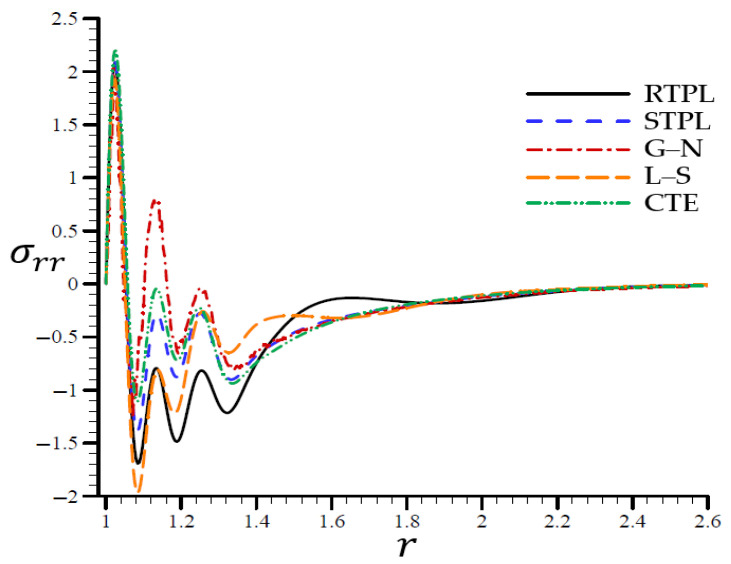
The radial stress $\sigma_{rr}$ through the radial direction of the spherical hole for all models.

**Figure 6:**
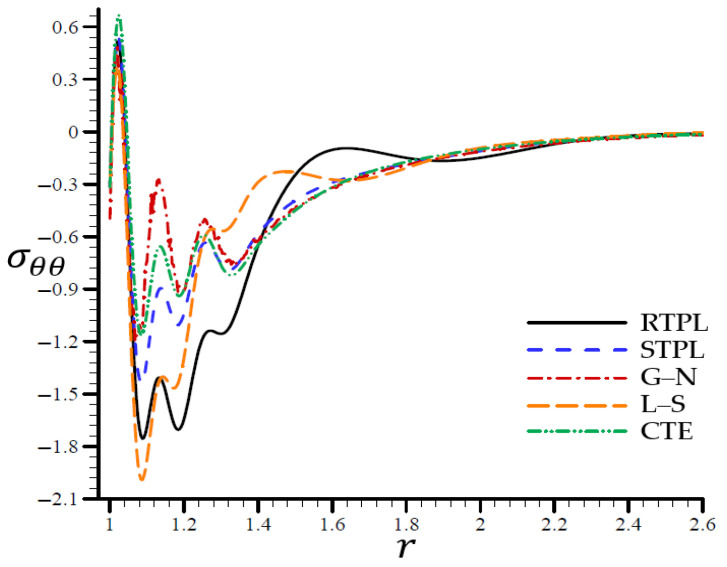
The circumferential stress $\sigma_{\theta \theta }$ through the radial direction of a spherical hole for all models.

**Figure 7:**
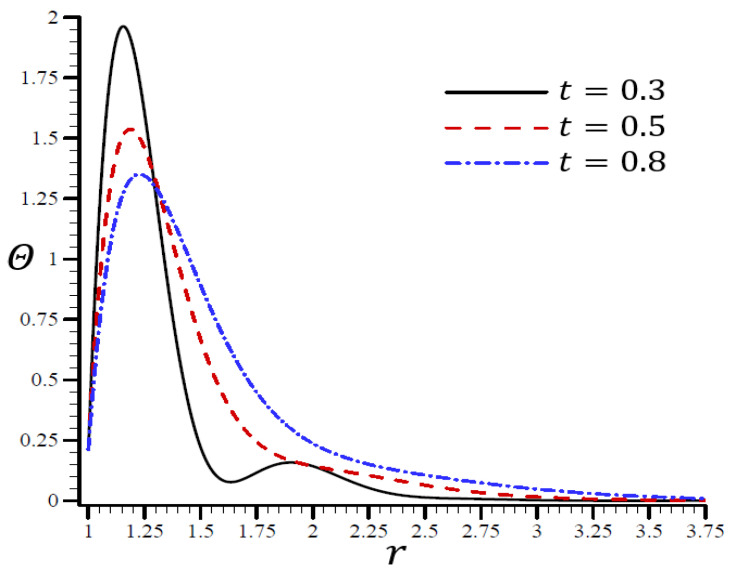
The influence of t on temperature Θ through the radial direction of a spherical hole using the RTPL model.

**Figure 8:**
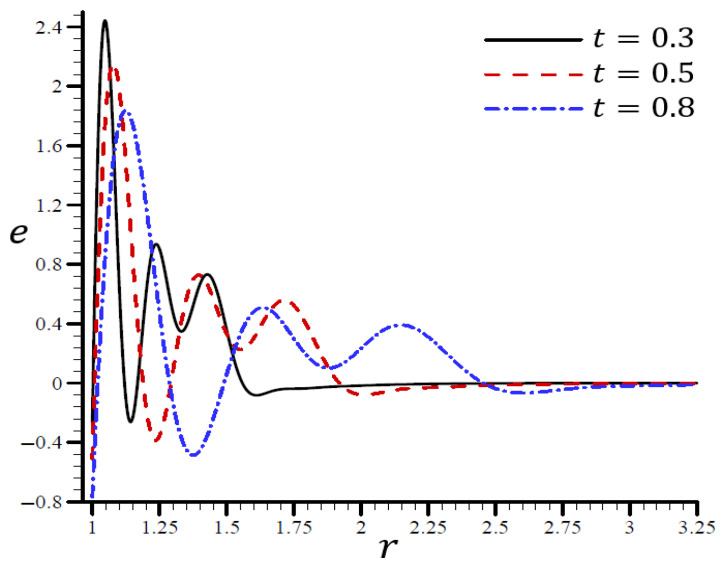
The influence of t on volumetric strain e through the radial direction of the spherical hole using the RTPL model.

**Figure 9:**
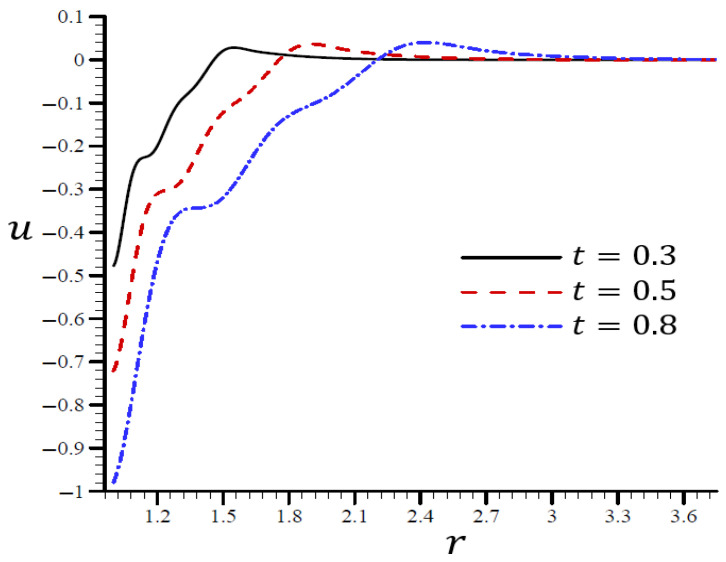
The influence of t on radial displacement u through the radial direction of the spherical hole using the RTPL model.

**Figure 10:**
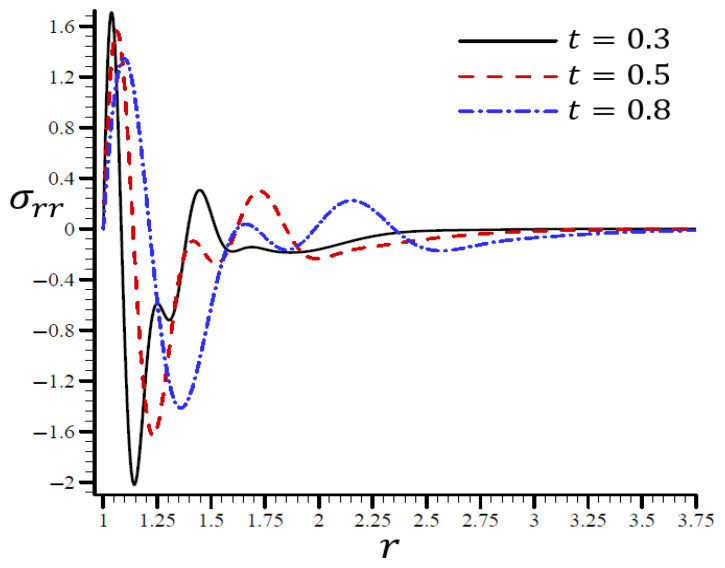
The influence of t on radial stress $\sigma_{rr}$ through the radial direction of the spherical hole using the RTPL model.

**Figure 11:**
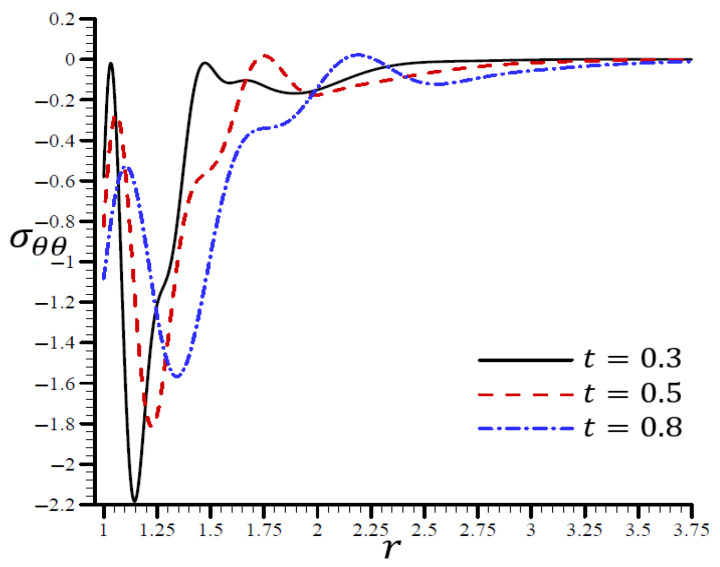
The influence of t on circumferential stress $\sigma_{\theta \theta }$ through the radial direction of the spherical hole using the RTPL model.

**Figure 12:**
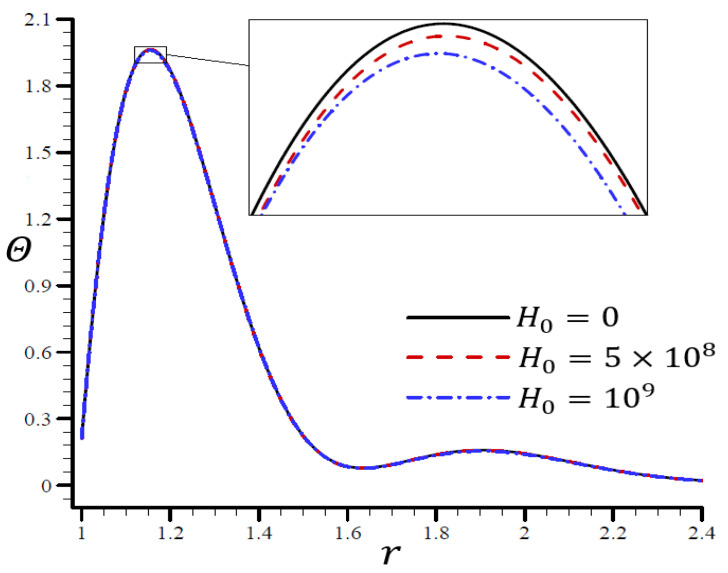
The influence of $H_{0}$ on temperature Θ through the radial direction of a spherical hole using the RTPL model.

**Figure 13:**
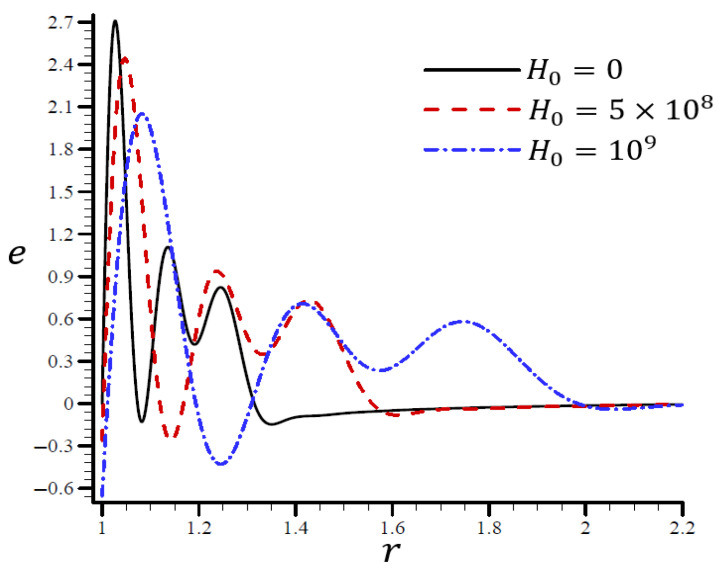
The influence of $H_{0}$ on volumetric strain e through the radial direction of the spherical hole using the RTPL model.

**Figure 14:**
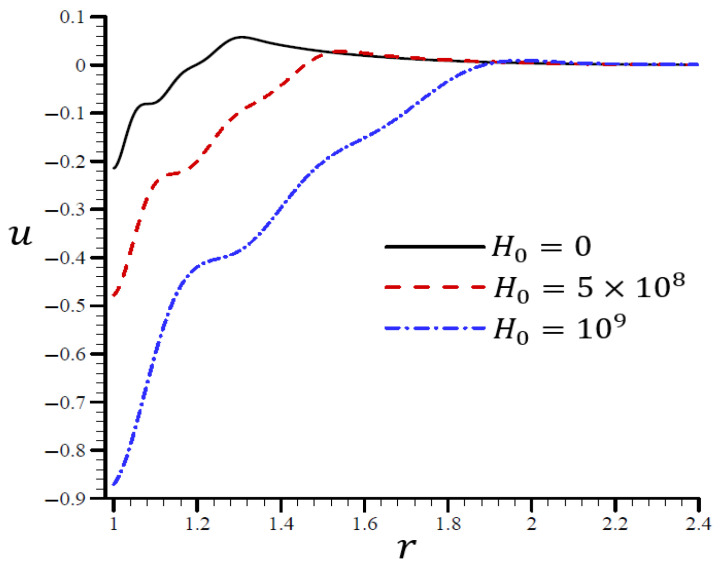
The influence of $H_{0}$ on radial displacement u through the radial direction of the spherical hole using the RTPL model.

**Figure 15:**
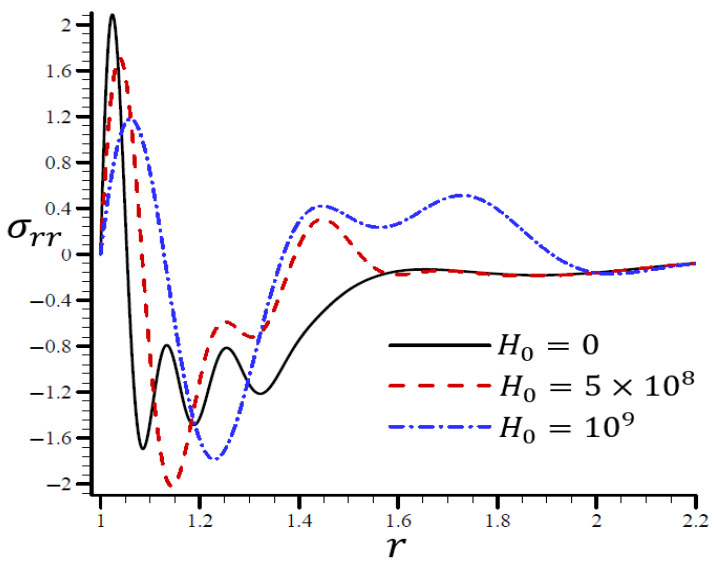
The influence of $H_{0}$ on radial stress $\sigma_{rr}$ through the radial direction of the spherical hole using the RTPL model.

**Figure 16:**
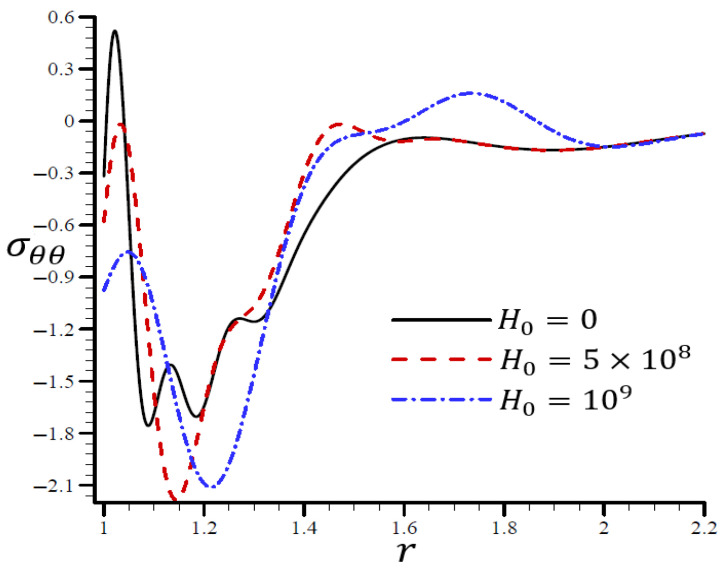
The influence of $H_{0}$ on circumferential stress $\sigma_{\theta \theta }$ through the radial direction of the spherical hole using the RTPL model.

**Figure 17:**
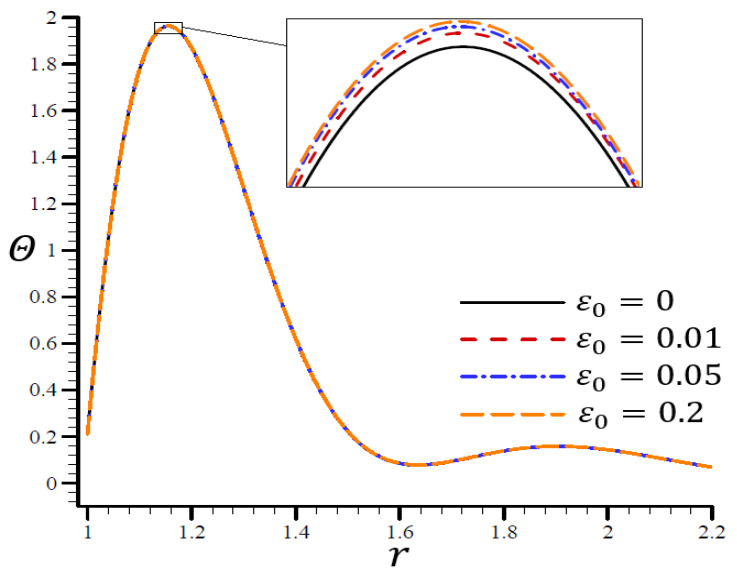
The influence of $\varepsilon_{0}$ on temperature Θ through the radial direction of a spherical hole using the RTPL model.

**Figure 18:**
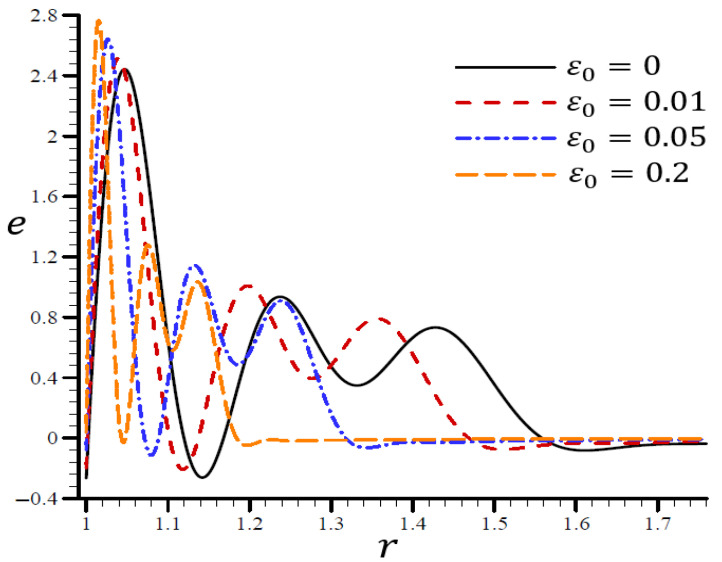
The influence of $\varepsilon_{0}$ on volumetric strain e through the radial direction of the spherical hole using the RTPL model.

**Figure 19:**
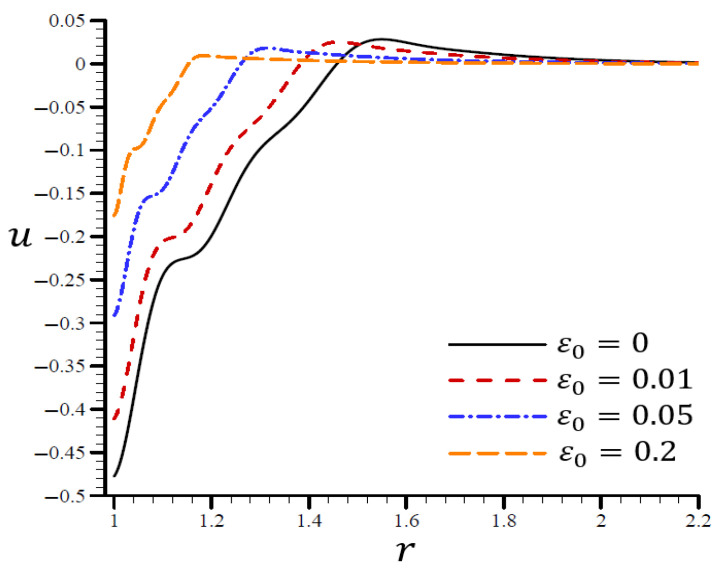
The influence of $\varepsilon_{0}$ on radial displacement u through the radial direction of the spherical hole using the RTPL model.

**Figure 20:**
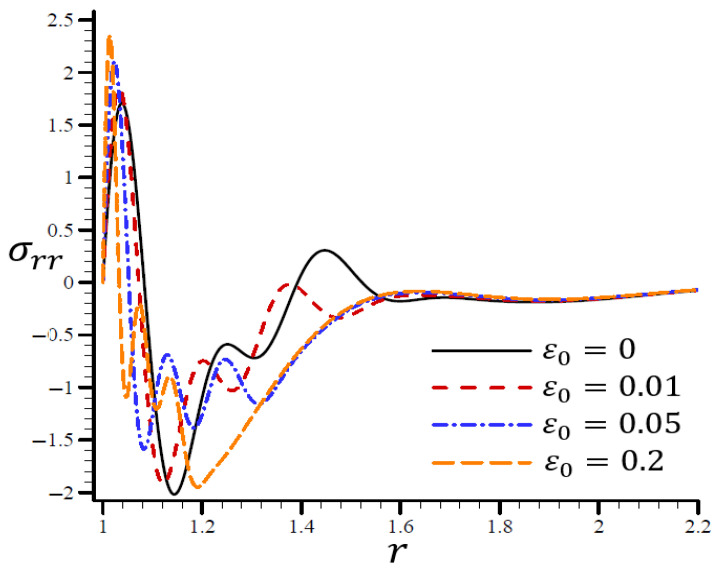
The influence of $\varepsilon_{0}$ on radial stress $\sigma_{rr}$ through the radial direction of the spherical hole using the RTPL model.

**Figure 21:**
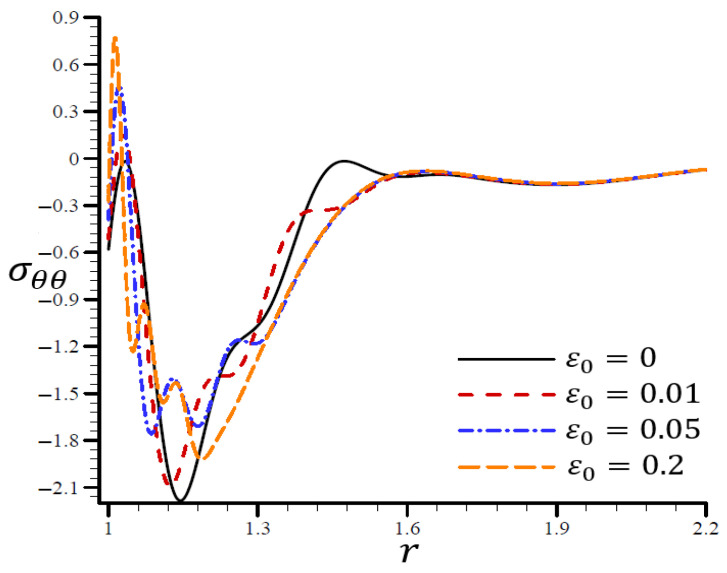
The influence of $\varepsilon_{0}$ on circumferential stress $\sigma_{\theta \theta }$ through the radial direction of the spherical hole using the RTPL model.

Table 1, Table 2, Table 3, Table 4, Table 5, Table 6, Table 7, Table 8, Table 9, Table 10, Table 11, Table 12, Table 13, Table 14 and Table 15 provide the results for all variables using various thermoelasticity models of triple-phase-lag in various locations. Each model’s field values were impacted by the magnetic field $H_{0}$ and electric permittivity $\varepsilon_{0}$ at a time-independent dimensionless rate of t=0.3. Figure 2, Figure 3, Figure 4, Figure 5, Figure 6, Figure 7, Figure 8, Figure 9, Figure 10, Figure 11, Figure 12, Figure 13, Figure 14, Figure 15, Figure 16, Figure 17, Figure 18, Figure 19, Figure 20 and Figure 21 show further results from an unbounded medium with a spherical hole in the radial direction. Except when otherwise noted, the numerical data in these tables were acquired for t=0.3, $\mu_{0}=1.256629\times 10^{-6}$, $H_{0}=5\times 10^{8}$, and $\varepsilon_{0}=8.85418782\times 10^{-12}$.

Table 1, Table 2, Table 3, Table 4, Table 5, Table 6, Table 7, Table 8, Table 9, Table 10, Table 11, Table 12, Table 13, Table 14 and Table 15 are inevitable to serve as standards for other studies. There are a lot of comparisons presented in such tables. The effects of the dimensionless time parameter t at some positions in the medium are shown in Table 1, Table 2, Table 3, Table 4 and Table 5. As t increased, the dilatation e, radial displacement u, and circumferential stress $\sigma_{\theta \theta }$ decreased at all positions. Distinct observations were noticed for the radial stress $\sigma_{rr}$. However, the temperature Θ decreased as t increased at the first position (r=1.02), while Θ increased as t increased at the third position (r=1.4).

The inclusion of the dimensionless magnetic field intensity $H_{0}$ at some positions in the medium are shown in Table 6, Table 7, Table 8, Table 9 and Table 10. As $H_{0}$ increased, the dilatation e, radial displacement u, and circumferential stress $\sigma_{\theta \theta }$ rapidly decreased at all positions. This may not occur for $\sigma_{\theta \theta }$ when r=1.4. At r=1.02, the radial stress $\sigma_{rr}$ decreased as $H_{0}$ increased while it increased at r=1.4. However, $\sigma_{rr}$ no longer increased at r=1.2 and decreased again when $H_{0}=10^{9}$. However, the temperature Θ slowly increased as $H_{0}$ increased at r=1.02 for different theories except for the G–N theory, in which Θ is still constant. At r=1.2, the temperature Θ due to CTE, L–S, and RTPL theories slightly decreased as $H_{0}$ increased Otherwise, and at r=1.2, Θ no longer decreased and increased again when $H_{0}=10^{9}$. Generally, the temperature Θ due to the G–N theory was still constant at all discussed positions.

Finally, in this respect, the inclusion of the dimensionless electric permittivity $\varepsilon_{0}$ at distinct positions in the medium are shown in Table 11, Table 12, Table 13, Table 14 and Table 15. It is clear that all variables were sensitive to the inclusion of $\varepsilon_{0}$.

According to the above-presented data, it is clear that:

1. The RTPL models were developed with N equal to 3, 4, and 5. Nevertheless, the STPL model was essentially provided when N=1.
2. Using the RTPL model, incredibly accurate results were generated.
3. The RTPL model yielded closed outcomes. All variables may be insensitive to larger values of N, particularly when N exceeds 5.
4. The magnetic field variables, which are the electric permittivity $\varepsilon_{0}$ and magnetic field intensity $H_{0}$, were taken into account to show their effects via all thermoelasticity theorems with various values and in different positions.

### 4.2. Second Justification

A time t=0.3 was used in Figure 2, Figure 3, Figure 4, Figure 5 and Figure 6 to demonstrate the effects of each model on the variables. Accordingly, all of the following graphs are shown about the refined triple-phase-lag (RTPL) model with N=5 and $H_{0}=50$, so that all field variables are examined.

Figure 2 depicts the temperature difference across the radial direction of a spherical hole matching all models. Figure 3, Figure 4, Figure 5 and Figure 6 show comparable figures for the remaining variables. For the CTE, L–S, G–N, and STPL models in Figure 2, the temperature oscillated along the trajectory of the RTPL model. After r=2.25, the temperature remained stable in all models, and the temperature values were near to one another.

According to the STPL, L–S, and CTE models in Figure 3, the e values follow the RTPL theory’s vibrational trajectory exactly. This model’s trajectory varies concerning the G–N model’s values of e. There was a noticeable shift in the e values after r=1.68, after which the values were similar to each other. In Figure 4, the CTE and STPL models have radial displacements u potentially identical to that of the RTPL model, which vanished in the direction of the radial motion. To put it another way, the G–N and L–S models’ displacements u may represent upper or lower limitations on the values produced by the RTPL model.

Figure 5 shows that the RTPL model radial stress $\sigma_{rr}$ vibrated with a large amplitude around the rest of the models from r=1.06 to r=1.8. Around this location, the radial stress levels oscillated around those predicted by the RTPL theory, although with a modest amplitude, until they cease after r=2.6.

Finally, Figure 6 shows similar behaviors of the circumferential stress as those of the radial stress. It shows that the RTPL model’s circumferential stress $\sigma_{\theta \theta }$ vibrated with a large amplitude around the rest of the models from r=1.06 to r=1.8. Around this location, the radial stress levels oscillated around those predicted by the RTPL theory, although with a modest amplitude, until they cease after r=2.6.

In brief, based on the data shown above, it can be determined that the RTPL model produces the most straightforward results. When it comes to this problem, we will use the RTPL theory to see how various parameters affect the field variables.

### 4.3. The Influence of Dimensionless Time

Figure 7, Figure 8, Figure 9, Figure 10 and Figure 11 show the results of the influence of dimensionless time t on all variables according to the RTPL model with $H_{0}=50$. Each variable yields a maximum value. With time, its immense worth has begun to diminish from its former heights.

Figure 7 clearly shows that for various values of t and different wavelengths, Θ vibrated in the radial direction. When r=1.14, the temperature Θ no longer increased and reached its maximum value. Regardless of the value of t, the temperature decreased as r increased.

It is shown in Figure 8 that the volumetric strain e vibrated in the radial direction of the spherical hole with distinct frequencies and varying amplitudes. An increase in time t led to an increase in the wavelength. When t=0.3, the volumetric strain e initially disappeared when r>1.55 and t=0.80. The volumetric strain e finally disappeared when r>3. When t=0.3 in Figure 9, the radial displacement u increased swiftly across the radial direction of the spherical hole but increased slowly when t=0.80.

Using various t values, Figure 10 shows how the RTPL model influences the radial stress $\sigma_{rr}$ in a spherical hole’s radial direction. Different wavelengths were used to describe the stress $\sigma_{rr}$, which increased in frequency as the time t increased. Figure 11 shows the circumferential stress $\sigma_{\theta \theta }$ in the radial direction of the spherical cavity using the RTPL model for various t values. Depending on the value of t, the circumferential stress vibrated at various wavelengths and became smoother as t increased. The radial and circumferential stresses, $\sigma_{rr}$ and $\sigma_{\theta \theta }$, disappeared as time progressed, as shown in the figures.

### 4.4. The Influence of Dimensionless Magnetic Field Intensity

Figure 12, Figure 13, Figure 14, Figure 15 and Figure 16 show the effects of the dimensionless magnetic field strength $H_{0}$ on all variables using the RTPL model. Figure 12 depicts the effects of $H_{0}$ on Θ along the radial direction of a spherical hole. Figure 13, Figure 14, Figure 15 and Figure 16 show comparable figures for the remaining variables. Figure 12 shows that the Θ vibrated in the radial direction for various values of $H_{0}$ and produced the same wavelengths with very slight differences, making them quite comparable.

Figure 13 shows that the volumetric strain e vibrated with multiple frequencies and varied amplitudes along the radial direction of the spherical hole. An increase in $H_{0}$ led to an increase in wavelength. When $H_{0}=0$, the volumetric strain e first dissipated when r>1.35 and $H_{0}=10^{9}$, and eventually disappeared when r>2. In Figure 14, when $H_{0}=0$, the radial displacement u increased rapidly throughout the radial direction of the spherical hole, but it increased slowly when $H_{0}=10^{9}$. As $H_{0}$ increased, the radial displacement u may have vanished.

Figure 15 illustrates the impact of the RTPL model on the radial stress $\sigma_{rr}$ in a spherical hole’s radial direction for a variety of $H_{0}$ values. Different wavelengths were employed to characterize the radial stress $\sigma_{rr}$, which increased in frequency as $H_{0}$ increased. Figure 16 depicts the circumferential stress $\sigma_{\theta \theta }$ in the radial direction of the spherical cavity as calculated by the RTPL model for a range of $H_{0}$ values. Depending on the $H_{0}$ value, the circumferential stress vibrated at different wavelengths and became increasingly smooth as $H_{0}$ increased. As shown by the graphs, the radial and circumferential stresses, denoted by $\sigma_{rr}$ and $\sigma_{\theta \theta }$, vanished as $H_{0}$ advanced.

### 4.5. The Influence of Dimensionless Electric Permittivity

The effects of dimensionless electric permittivity $\varepsilon_{0}$ on all variables, as predicted by the RTPL model ($H_{0}=50$), are depicted in Figure 17, Figure 18, Figure 19, Figure 20 and Figure 21. The effects of $\varepsilon_{0}$ on Θ in the radial direction of a spherical hole are shown in Figure 17. Figure 18, Figure 19, Figure 20 and Figure 21 provide analogous data for the other variables. Figure 17 demonstrates that the Θ vibrated in the radial direction for a variety of $\varepsilon_{0}$ values and exhibited wavelengths that are almost identical with minor variations, making them similar.

According to Figure 18, the spherical hole’s volumetric strain e vibrated in the radial direction with different frequencies and varying amplitudes. The wavelength decreased as $\varepsilon_{0}$ increased. In both cases, when $\varepsilon_{0}=0$, the volumetric strain e initially reduced at r>1.7 and $\varepsilon_{0}=0.2$, and finally vanished at r>1.22. $\varepsilon_{0}$ increased the maximum value of e and became too close to the boundary layer, making $\varepsilon_{0}$ a more desirable parameter. On the spherical hole in Figure 19, the radial displacement u increased slowly when $\varepsilon_{0}=0$ and quickly when $\varepsilon_{0}=0.2$. As $\varepsilon_{0}$ grew, u vanished.

A range of $\varepsilon_{0}$ values are shown in Figure 20 to illustrate the effect of the RTPL model on the radial stress $\sigma_{rr}$ in a spherical hole’s radial direction. A wide range of wavelengths was used to study how radial stress $\sigma_{rr}$ changed with increasing values of $\varepsilon_{0}$. Figure 21 shows the circumferential stress $\sigma_{\theta \theta }$, as determined by an iterative application of the RTPL model, in a spherical hole’s radial direction with varied $\varepsilon_{0}$ values. The circumferential stress vibrated at different frequencies depending on the value of $\varepsilon_{0}$. It can be seen from the figures that as $\varepsilon_{0}$ increased, the radial and circumferential stresses, $\sigma_{rr}$ and $\sigma_{\theta \theta }$, were diminished, but their maximum values increased.

## 5. Conclusions

The new revised triple-phase-lag model is innovative and precise in terms of temperature, volumetric strain, displacement, and stresses. The heat equation with multi-time derivatives was explained. The thermoelastic coupling behavior of an infinite medium with a spherical cavity due to uniform heat was studied using spherical coordinates. The paired dynamical thermoelasticity model, the Lord and Shulman model, the Green and Naghdi model without energy dissipation, and a simple triple-phase-lag model were used to create a unified model. As a result of solving the two high-order time-derivative differential coupled equations, the thermoelastic coupling response of an infinite material with a spherical hole was produced. Several examples and applications were provided to compare the results of all models, regardless of whether or not they were subject to Lorentz Forces effects. As an example, a spherical hole was used to show the correlations between several factors. Tables have been supplied to serve as benchmarks for future comparisons by other scholars, as shown in the following instances. The disclosed and verified outcomes revealed that all field variables and dimensionless temporal parameters behaved differently from what had been assumed beforehand. Because of the triple-phase-lag theory, the magnitudes of several variables may be reduced in practical implementations. As long as the L–S model was used, the results were accurate. In contrast, the updated model yielded more precise results.

## Figures and Tables

**Table 1:** Different thermoelasticity theories with a range of *r* values show the effects of dimensionless time *t* on volumetric strain *e*.

*r*	*t*	CTE	G–N	L–S	STPL	RTPL		
					*N* = 1	*N* = 3	*N* = 4	*N* = 5
1.001	0.3	−0.1600507	−0.1103681	−0.1550835	−0.1590269	−0.1588199	−0.1596750	−0.1596267
	0.5	−0.4421455	−0.4275114	−0.4390305	−0.4434662	−0.4443045	−0.4443921	−0.4443562
	0.8	−0.7204259	−0.7270958	−0.7191311	−0.7256527	−0.7265913	−0.7265860	−0.7265783
1.0108	0.3	0.7907324	0.9283637	0.8158773	0.7976376	0.8076523	0.8034979	0.8027793
	0.5	0.1513132	0.1735829	0.1667559	0.1553263	0.1569277	0.1563401	0.1563651
	0.8	−0.3411962	−0.3475165	−0.3323632	−0.3422239	−0.3425316	−0.3425963	−0.3425864
1.035	0.3	2.2338375	2.8512535	2.2802879	2.2427386	2.2771291	2.2703739	2.2670290
	0.5	1.3241083	1.4192143	1.3607036	1.3359762	1.3456487	1.3440881	1.3439886
	0.8	0.4858891	0.4793232	0.5099324	0.4931183	0.4951689	0.4949403	0.4949480

**Table 2:** Different thermoelasticity theories with a range of *r* values show the effects of dimensionless time *t* on radial displacement *u*.

*r*	*t*	CTE	G–N	L–S	STPL	RTPL		
					*N* = 1	*N* = 3	*N* = 4	*N* = 5
1.02	0.3	−0.4446010	−0.4587127	−0.4416027	−0.4442389	−0.4446845	−0.4452007	−0.4451150
	0.5	−0.6879661	−0.6641208	−0.6859852	−0.6897589	−0.6907317	−0.6907757	−0.6907412
	0.8	−0.94270823	−0.9609906	−0.9420769	−0.9482101	−0.9491696	−0.9491588	−0.9491515
1.2	0.3	−0.1990386	−0.1923100	−0.1999577	−0.1995811	−0.1997097	−0.1990796	−0.1991871
	0.5	−0.3095016	−0.3218320	−0.3080032	−0.3111561	−0.3096710	−0.3097699	−0.3098091
	0.8	−0.4649046	−0.4700720	−0.4608963	−0.4675405	−0.4668571	−0.4669065	−0.4669071
1.4	0.3	−0.0421606	−0.0347817	−0.0412343	−0.0414258	−0.0426042	−0.0424287	−0.0422553
	0.5	−0.1976639	−0.1892863	−0.1990575	−0.1983774	−0.1989738	−0.1988528	−0.1988503
	0.8	−0.3399756	−0.3397367	−0.3428069	−0.3434390	−0.3428693	−0.3428636	−0.3428703

**Table 3:** Different thermoelasticity theories with a range of *r* values show the effects of dimensionless time *t* on temperature Θ.

*r*	*t*	CTE	G–N	L–S	STPL	RTPL		
					*N* = 1	*N* = 3	*N* = 4	*N* = 5
1.02	0.3	0.5197161	0.5231952	0.8158523	0.6321797	0.7257061	0.6687072	0.6619560
	0.5	0.4551331	0.4591944	0.6034615	0.5250257	0.5347617	0.5288343	0.5292230
	0.8	0.4065923	0.4113274	0.4823044	0.4480926	0.4475421	0.4469535	0.4470211
1.2	0.3	1.1328376	1.1551850	1.5158575	1.2732250	1.8693517	1.8697841	1.7797076
	0.5	1.1441023	1.1762359	1.6486669	1.3572497	1.5595642	1.5331112	1.5294970
	0.8	1.0991674	1.1409540	1.4633732	1.2932336	1.3452712	1.3412060	1.3411794
1.4	0.3	0.6166223	0.6403425	0.2553162	0.5498148	0.4213529	0.6197291	0.6189961
	0.5	0.7944132	0.8302729	0.7959542	0.8370789	0.9866523	0.9826821	0.9777945
	0.8	0.9145463	0.9654823	1.1009292	1.0413519	1.1155064	1.112294	1.1119570

**Table 4:** Different thermoelasticity theories with a range of r values show the effects of dimensionless time t on radial stress $\sigma_{rr}$.

*r*	*t*	CTE	G–N	L–S	STPL	RTPL		
					*N* = 1	*N* = 3	*N* = 4	*N* = 5
1.02	0.3	1.4250082	1.7169427	1.1644764	1.3218307	1.2488604	1.3002398	1.3052216
	0.5	0.8697574	0.8919956	0.7447060	0.8095222	0.8052005	0.8101600	0.8097287
	0.8	0.5084246	0.5016690	0.4473304	0.4747826	0.4767497	0.4771983	0.4771340
1.2	0.3	−0.3476186	−0.2060406	−0.7381048	−0.4810923	−1.1013084	−1.0979774	−1.0048155
	0.5	−1.0358693	−0.4342299	−1.5929489	−1.2634836	−1.4691980	−1.4406952	−1.4372923
	0.8	0.4730670	0.4169757	0.0996810	0.2792902	0.2373867	0.2412938	0.2412457
1.4	0.3	0.0720812	−0.0097442	0.4509219	0.1469006	0.2787176	0.0756354	0.0768349
	0.5	0.0635672	0.0801358	0.0794198	0.0336441	−0.1175433	−0.1139438	−0.1088892
	0.8	−1.1153279	−1.1761286	−1.3292837	−1.2426448	−1.3245219	−1.3209060	−1.3205495

**Table 5:** Different thermoelasticity theories with a range of r values show the effects of dimensionless time t on circumferential stress $\sigma_{\theta \theta }$.

*r*	*t*	CTE	G–N	L–S	STPL	RTPL		
					*N* = 1	*N* = 3	*N* = 4	*N* = 5
1.02	0.3	0.0198399	0.1669378	−0.2555124	−0.0876141	−0.1712722	−0.1175958	−0.1116476
	0.5	−0.4645790	−0.4416541	−0.5992993	−0.5313858	−0.5393601	−0.5339610	−0.5343373
	0.8	−0.8709283	−0.8892813	−0.9386944	−0.9138763	−0.9135551	−0.9130260	−0.9130849
1.2	0.3	−0.9048642	−0.8304659	−1.2923918	−1.0422374	−1.6505469	−1.6485684	−1.5570346
	0.5	−1.3474299	−1.0602676	−1.8770728	−1.5692064	−1.7719893	−1.7445910	−1.7411154
	0.8	−0.6979375	−0.7436193	−1.0634094	−0.8940525	−0.9404411	−0.9364963	−0.9365076
1.4	0.3	−0.3014565	−0.3536854	0.0693004	−0.2301085	−0.1008053	−0.3014154	−0.3003248
	0.5	−0.5053201	−0.5032281	−0.4991360	−0.5421070	−0.6929150	−0.6890443	−0.6840712
	0.8	−1.2577217	−1.3109739	−1.4599464	−1.3872542	−1.4648736	−1.4614548	−1.4611128

**Table 6:** Different thermoelasticity theories with a range of r values show the effects of dimensionless magnetic field intensity $H_{0}$ on volumetric strain e.

*r*	*H*_0_	CTE	G–N	L–S	STPL	RTPL		
					*N* = 1	*N* = 3	*N* = 4	*N* = 5
1.001	0	0.1782137	0.5045876	0.1876537	0.1803101	0.1815128	0.1801641	0.1801591
	$5\times 10^{8}$	−0.1600507	−0.1260185	−0.1550835	−0.1590269	−0.1588199	−0.1596750	−0.1596267
	$1\times 10^{9}$	−0.5963013	−0.5838559	−0.5942398	−0.5959014	−0.5961448	−0.5966622	−0.5965978
1.0108	0	1.6863479	3.1787268	1.7322308	1.6976456	1.7199434	1.7128199	1.7110496
	$5\times 10^{8}$	0.7907324	0.9125334	0.8158773	0.7976376	0.8076523	0.8034979	0.8027793
	$1\times 10^{9}$	−0.0216654	−0.0121308	−0.0097624	−0.0180902	−0.0141696	−0.0163392	−0.0165856
1.035	0	2.4534254	7.7106569	2.4972843	2.4545775	2.5100973	2.5053423	2.4991835
	$5\times 10^{8}$	2.2338375	2.8364917	2.2802879	2.2427386	2.2771291	2.2703739	2.2670290
	$1\times 10^{9}$	1.1346513	1.1417442	1.1625329	1.1418607	1.1575618	1.1530407	1.1516357

**Table 7:** Different thermoelasticity theories with a range of r values show the effects of dimensionless magnetic field intensity $H_{0}$ on radial displacement u.

r	$H_{0}$	CTE	G–N	L–S	STPL	RTPL		
					N=1	N=3	N=4	N=5
1.02	0	−0.1767007	−0.2365661	−0.1712572	−0.1759073	−0.1761001	−0.1767683	−0.1766927
	$5\times 10^{8}$	−0.4446010	−0.4556610	−0.4416027	−0.4442389	−0.4446845	−0.4452007	−0.4451150
	$1\times 10^{9}$	−0.8377249	−0.8346584	−0.8365695	−0.8376432	−0.8381650	−0.8385221	−0.8384422
1.2	0	0.0006626	0.0211928	0.0018281	0.0009625	0.0010703	0.0015785	0.0014699
	$5\times 10^{8}$	−0.1990386	−0.1927053	−0.1999577	−0.1995811	−0.1997097	−0.1990796	−0.1991871
	$1\times 10^{9}$	−0.4200627	−0.4180902	−0.4204997	−0.4207381	−0.4193394	−0.4190292	−0.4192868
1.4	0	0.0415410	0.0465105	0.0418797	0.0420817	0.0412667	0.0413940	0.0415588
	$5\times 10^{8}$	−0.0421606	−0.0352435	−0.0412343	−0.0414258	−0.0426042	−0.0424287	−0.0422553
	$1\times 10^{9}$	−0.2954475	−0.2962609	−0.2956049	−0.2953487	−0.2970595	−0.2968102	−0.2965584

**Table 8:** Different thermoelasticity theories with a range of r values show the effects of dimensionless magnetic field intensity $H_{0}$ on temperature Θ.

r	$H_{0}$	CTE	G–N	L–S	STPL	RTPL		
					N=1	N=3	N=4	N=5
1.02	0	0.5195277	0.5231952	0.8149122	0.6317500	0.7249938	0.6681243	0.6613913
	$5\times 10^{8}$	0.5197161	0.5231952	0.8158523	0.6321797	0.7257061	0.6687072	0.6619559
	$1\times 10^{9}$	0.5202053	0.5231952	0.8171815	0.6329834	0.7267366	0.6695850	0.6628219
1.2	0	1.1328485	1.1551850	1.5160141	1.2730366	1.8699681	1.8702848	1.7801007
	$5\times 10^{8}$	1.1328376	1.1551850	1.5158575	1.2732250	1.8693517	1.8697841	1.7797076
	$1\times 10^{9}$	1.1302030	1.1551850	1.5072709	1.2688359	1.8634498	1.8652445	1.7751423
1.4	0	0.6178070	0.6403425	0.2557158	0.5506273	0.4221051	0.6206186	0.6198641
	$5\times 10^{8}$	0.6166223	0.6403425	0.2553162	0.5498148	0.4213529	0.6197291	0.6189961
	$1\times 10^{9}$	0.6177501	0.6403425	0.2597756	0.5516250	0.4242953	0.6217558	0.6210694

**Table 9:** Different thermoelasticity theories with a range of r values show the effects of dimensionless magnetic field intensity $H_{0}$ on radial stress $\sigma_{rr}$.

r	$H_{0}$	CTE	G–N	L–S	STPL	RTPL		
					N=1	N=3	N=4	N=5
1.02	0	2.1413094	5.0084304	1.9012246	2.0400614	1.9882193	2.0368781	2.0397306
	$5\times 10^{8}$	1.4250082	1.7098567	1.1644764	1.3218307	1.2488604	1.3002398	1.3052216
	$1\times 10^{9}$	0.7663735	0.7619035	0.4877997	0.6591606	0.5742749	0.6284021	0.6344444
1.2	0	−0.6773446	0.6615173	−1.0776863	−0.8119883	−1.4290790	−1.4239547	−1.3317608
	$5\times 10^{8}$	−0.3476186	−0.2009060	−0.7381048	−0.4810923	−1.1013084	−1.0979774	−1.0048155
	$1\times 10^{9}$	−0.8607713	−0.8969050	−1.2863322	−1.0105215	−1.6267403	−1.6169840	−1.5257144
1.4	0	−0.7407481	−0.7747852	−0.3827128	−0.6759330	−0.5439698	−0.7468172	−0.7458980
	$5\times 10^{8}$	0.0720812	−0.0016496	0.4509219	0.1469006	0.2787176	0.0756354	0.0768349
	$1\times 10^{9}$	0.2771069	0.2558551	0.6657660	0.3510599	0.4892780	0.2833072	0.2842716

**Table 10:** Different thermoelasticity theories with a range of r values show the effects of dimensionless magnetic field intensity $H_{0}$ on circumferential stress $\sigma_{\theta \theta }$.

r	$H_{0}$	CTE	G–N	L–S	STPL	RTPL		
					N=1	N=3	N=4	N=5
1.02	0	0.6413170	1.9878850	0.3789836	0.5353730	0.4626946	0.5147940	0.5196557
	$5\times 10^{8}$	0.0198399	0.1372332	−0.2555124	−0.0876141	−0.1712722	−0.1175958	−0.1116476
	$1\times 10^{9}$	−0.6954887	−0.6792086	−0.9821086	−0.8053970	−0.8952159	−0.8399301	−0.8334501
1.2	0	−0.9039566	−0.2244379	−1.2947625	−1.0411157	−1.6480632	−1.6452294	−1.5541281
	$5\times 10^{8}$	−0.9048642	−0.8273155	−1.2923918	−1.0422374	−1.6505469	−1.6485684	−1.5570346
	$1\times 10^{9}$	−1.3447378	−1.3652770	−1.7464786	−1.4895059	−2.0937861	−2.0895322	−1.9990591
1.4	0	−0.6498027	−0.6794221	−0.2895031	−0.5834226	−0.4537569	−0.6543521	−0.6533975
	$5\times 10^{8}$	−0.3014565	−0.3479797	0.0693004	−0.2301085	−0.1008053	−0.3014154	−0.3003248
	$1\times 10^{9}$	−0.3799260	−0.4038305	−0.0066819	−0.3098064	−0.1782388	−0.3797877	−0.3787822

**Table 11:** Different thermoelasticity theories with a range of r values show the effects of dimensionless electric permittivity $\varepsilon_{0}$ on volumetric strain e.

r	$\varepsilon_{0}$	CTE	G–N	L–S	STPL	RTPL		
					N=1	N=3	N=4	N=5
1.001	0.00	−0.1600507	−0.1817118	−0.1550835	−0.1590269	−0.1588199	−0.1596750	−0.1596267
	0.01	−0.0728988	−0.2495945	−0.0685479	−0.0719793	−0.0716577	−0.0723496	−0.0723247
	0.05	0.1069584	0.5070073	0.1099905	0.1076340	0.1080314	0.1076008	0.1075979
	0.20	0.3598124	−0.1230445	0.3614521	0.3602013	0.3605292	0.3603088	0.3602955
1.0108	0.00	0.7907324	0.7422693	0.8158773	0.7976376	0.8076523	0.8034979	0.8027793
	0.01	1.0444167	1.1310202	1.0660570	1.0501743	1.0593502	1.0558573	1.0551759
	0.05	1.6586577	2.2876971	1.6733614	1.6622524	1.6694769	1.6672005	1.6666240
	0.20	2.5373701	−0.1390619	2.5446030	2.5388002	2.5434033	2.5423482	2.5419391
1.035	0.00	2.2338375	2.0604843	2.2802879	2.2427386	2.2771291	2.2703739	2.2670290
	0.01	2.4541706	3.6055484	2.4889375	2.4596437	2.4888941	2.4840987	2.4811503
	0.05	2.3896894	3.7194494	2.4028985	2.3898009	2.4073189	2.4059421	2.4039812
	0.20	0.5555053	−3.5966438	0.5537322	0.5538753	0.5579398	0.5586249	0.5580035

**Table 12:** Different thermoelasticity theories with a range of r values show the effects of dimensionless electric permittivity $\varepsilon_{0}$ on radial displacement u.

r	$\varepsilon_{0}$	CTE	G–N	L–S	STPL	RTPL		
					N=1	N=3	N=4	N=5
1.02	0.00	−0.4446010	−0.4702098	−0.4416027	−0.4442389	−0.4446845	−0.4452007	−0.4451150
	0.01	−0.3761698	−0.3938417	−0.3735556	−0.3758274	−0.3760925	−0.3764870	−0.3764284
	0.05	−0.2514169	−0.2002410	−0.2496761	−0.2511617	−0.2512163	−0.2514271	−0.2514038
	0.20	−0.1285389	−0.1350768	−0.1277263	−0.1284124	−0.1283882	−0.1284682	−0.1284637
1.2	0.00	−0.1990386	−0.1982898	−0.1999577	−0.1995811	−0.1997097	−0.1990796	−0.1991871
	0.01	−0.1388393	−0.1242475	−0.1387854	−0.1389520	−0.1390226	−0.1386521	−0.1387124
	0.05	−0.0514207	−0.0617681	−0.0510383	−0.0513124	−0.0512864	−0.0511259	−0.0511593
	0.20	0.0085746	0.0088881	0.0087642	0.0086495	0.0086748	0.0087255	0.0087156
1.4	0.00	−0.0421606	−0.0399129	−0.0412343	−0.0414258	−0.0426042	−0.0424287	−0.0422553
	0.01	0.0104176	0.0181003	0.0115143	0.0111229	0.0104788	0.0105647	0.0106848
	0.05	0.0128624	0.0116589	0.0129452	0.0130233	0.0127674	0.0128069	0.0128582
	0.20	0.0039736	0.0026442	0.0039234	0.0039991	0.0039099	0.0039224	0.0039389

**Table 13:** Different thermoelasticity theories with a range of r values show the effects of dimensionless electric permittivity $\varepsilon_{0}$ on temperature Θ.

r	$\varepsilon_{0}$	CTE	G–N	L–S	STPL	RTPL		
					N=1	N=3	N=4	N=5
1.02	0.00	0.5197161	0.5231952	0.8158523	0.6321797	0.7257061	0.6687072	0.6619559
	0.01	0.5196226	0.5231952	0.8155291	0.6320045	0.7254572	0.6684988	0.6617520
	0.05	0.5195045	0.5231952	0.8149457	0.6317351	0.7250108	0.6681320	0.6613971
	0.20	0.5194992	0.5231952	0.8144369	0.6315960	0.7246144	0.6678177	0.6611030
1.2	0.00	1.1328376	1.1551850	1.5158575	1.2732250	1.8693517	1.8697841	1.7797076
	0.01	1.1325365	1.1551850	1.5158537	1.2728365	1.8696724	1.8700076	1.7798489
	0.05	1.1326225	1.1551850	1.5155747	1.2727896	1.8697239	1.8700990	1.7799023
	0.20	1.1330736	1.1551850	1.5160089	1.2731337	1.8702705	1.8706068	1.7803877
1.4	0.00	0.6166223	0.6403425	0.2553162	0.5498148	0.4213529	0.6197291	0.6189961
	0.01	0.6163464	0.6403425	0.2546471	0.5492825	0.4209161	0.6193444	0.6186026
	0.05	0.6175008	0.6403425	0.2554513	0.5503468	0.4216919	0.6202147	0.6194853
	0.20	0.6182038	0.6403425	0.2561957	0.5510730	0.4223512	0.6209149	0.6201905

**Table 14:** Different thermoelasticity theories with a range of r values show the effects of dimensionless electric permittivity $\varepsilon_{0}$ on radial stress $\sigma_{rr}$.

r	$\varepsilon_{0}$	CTE	G–N	L–S	STPL	RTPL		
					N=1	N=3	N=4	N=5
1.02	0.00	1.4250082	1.4417132	1.1644764	1.3218307	1.2488604	1.3002398	1.3052216
	0.01	1.6799725	2.3462039	1.4135395	1.5747718	1.4996322	1.5520136	1.5571447
	0.05	2.1955956	2.8931530	1.9176461	2.0867605	2.0068048	2.0610981	2.0665780
	0.20	2.1293602	−1.9570943	1.8395231	2.0174381	1.9310497	1.9872334	1.9932340
1.2	0.00	−0.3476186	−0.7002070	−0.7381048	−0.4810923	−1.1013084	−1.0979774	−1.0048155
	0.01	−0.0084140	0.7900171	−0.3912182	−0.1439469	−0.7489823	−0.7479519	−0.6564771
	0.05	−0.5214154	0.2873304	−0.9091673	−0.6595318	−1.2628424	−1.2615757	−1.1707216
	0.20	−1.1843148	−3.7578587	−1.5710519	−1.3248846	−1.9236769	−1.9235961	−1.8331840
1.4	0.00	0.0720812	−0.0630823	0.4509219	0.1469006	0.2787176	0.0756354	0.0768349
	0.01	−0.0733074	−0.1260033	0.2946380	−0.0050862	0.1296550	−0.0721929	−0.0714200
	0.05	−0.6513165	−0.4203154	−0.2904336	−0.5848771	−0.4551246	−0.6549940	−0.6542153
	0.20	−0.6302124	−1.3114760	−0.2682290	−0.5632011	−0.4341115	−0.6331041	−0.6323633

**Table 15:** Different thermoelasticity theories with a range of r values show the effects of dimensionless electric permittivity $\varepsilon_{0}$ on circumferential stress $\sigma_{\theta \theta }$.

r	$\varepsilon_{0}$	CTE	G–N	L–S	STPL	RTPL		
					N=1	N=3	N=4	N=5
1.02	0.00	0.0198399	−0.1007279	−0.2555124	−0.0876141	−0.1712722	−0.1707365	−0.1706840
	0.01	0.2147006	0.3924400	−0.0638714	0.1062539	0.0217218	0.0222527	0.0223048
	0.05	0.5953855	1.0740053	0.3104173	0.4851070	0.3984551	0.3989805	0.3990321
	0.20	0.6824975	−1.5054263	0.3909123	0.5706121	0.4809410	0.4814663	0.4815179
1.2	0.00	−0.9048642	−1.1056940	−1.2923918	−1.0422374	−1.6505469	−1.6485537	−1.6483581
	0.01	−0.6845724	−0.3075093	−1.0675876	−0.8225765	−1.4235815	−1.4215722	−1.4213750
	0.05	−0.8690243	−0.4528431	−1.2540643	−1.0080733	−1.6081824	−1.6061455	−1.6059456
	0.20	−1.1516241	−2.2032886	−1.5363075	−1.2918775	−1.8898230	−1.8877660	−1.8875641
1.4	0.00	−0.3014565	−0.3782174	0.0693004	−0.2301085	−0.1008053	−0.0993506	−0.0992079
	0.01	−0.3366938	−0.3633738	0.0289190	−0.2685467	−0.1374440	−0.1359819	−0.1358384
	0.05	−0.6252768	−0.5124963	−0.2637530	−0.5583662	−0.4293436	−0.4278723	−0.4277280
	0.20	−0.6213890	−0.9706441	−0.2594291	−0.5542999	−0.4254574	−0.4239819	−0.4238371

## Data Availability

Not applicable.
